# Sexual dysfunction and satisfaction in obsessive compulsive disorder: protocol for a systematic review and meta-analysis

**DOI:** 10.1186/s13643-019-1262-7

**Published:** 2020-01-09

**Authors:** Andrea Pozza, David Veale, Donatella Marazziti, Jaime Delgadillo, Umberto Albert, Giacomo Grassi, Davide Prestia, Davide Dèttore

**Affiliations:** 1 Department of Medical Sciences, Surgery and Neurosciences, University of Siena, Siena, Italy; 2 Institute of Psychiatry, Psychology and Neurosciences, King’s College London, London, UK; 3 South London and Maudsley, NHS Trust, London, UK; 4 Department of Clinical and Experimental Medicine, Section of Psychiatry, University of Pisa, Pisa, Italy; 5 Clinical Psychology Unit, Department of Psychology, University of Sheffield, Sheffield, UK; 6 Department of Medicine, Surgery and Health Sciences, University of Trieste, Trieste, Italy; 7 Brain Center Firenze, Florence, Italy; 8 Department of Neuroscience, Rehabilitation, Opthalmology, Genetics, Maternal and Child Health (DINOGMI), Section of Psychiatry, University of Genoa IRCCS San Martino, Genoa, Italy; 9 Department of Health Sciences, University of Florence, Florence, Italy

**Keywords:** Obsessive compulsive disorder, Sexual dysfunction, Sexual satisfaction, Sexual desire, Orgasm, Excitation, Arousal, Well-being, Quality of life, Systematic review

## Abstract

**Background:**

Obsessive compulsive disorder (OCD) is a chronic mental health condition recognized as one of the most serious causes of disability and impaired quality of life. In the literature, there is no review about sexual dysfunction and satisfaction in OCD. The current paper presents the protocol for a systematic review and meta-analysis aimed to summarize data (1) comparing the presence of sexual dysfunction between groups with OCD and non-clinical groups, (2) investigating prevalence of each one of the sexual dysfunctions in patients with OCD, (3) comparing risk for sexual dysfunction in OCD groups with the prevalence in control groups, (4) comparing sexual satisfaction between OCD groups and non-clinical groups, and (5) investigating moderators of sexual dysfunction in OCD groups as compared with control groups. Gender, age, marital status, OCD symptom severity and subtypes, comorbid depressive disorders, comorbid anxiety disorders, concurrent psychiatric medications, comorbid general medical disease, and study quality will be investigated as moderators.

**Methods:**

The protocol is reported according to PRISMA-P guidelines. The search will be conducted by independent reviewers during the second week of December 2019 by using electronic databases (Scopus, PubMed, EMBASE, PsycINFO, CINAHL, and the Cochrane Library), by contacting the authors of the included studies to identify further data, by examining the references of the included studies, and by handsearching conference proceedings and theses/doctoral dissertations. The study quality will be independently evaluated using the Newcastle-Ottawa Quality Assessment Scale. Random-effect meta-analyses will be computed. If there is insufficient data for a specific outcome, only a systematic review will be performed.

**Discussion:**

This review may support clinical practice highlighting the importance of the assessment of sexuality in patients with OCD and suggesting the use of therapeutic strategies dedicated to sexuality in this clinical population with the aim of improving patients’ quality of life. Potential limitations will regard the heterogeneity of the studies in terms of the instruments used to assess sexual dysfunction/satisfaction and of the definitions used to conceptualize sexual dysfunction.

**Systematic review registration:**

Prospero CRD42019132264

## Background

### Clinical picture and treatment challenges of OCD

Obsessive compulsive disorder (OCD) is a chronic mental health condition characterized by recurrent unwanted thoughts, impulses, or mental images called obsessions, accompanied by repetitive behaviours called compulsions [1]. While obsessions provoke unpleasant emotions including anxiety, disgust, or guilt, compulsions are repetitively performed to cope with the unpleasant feelings evoked by the former, although relief is often only temporary [1]. This condition has a 2–3% lifetime prevalence and an equal gender distribution in the general population [2]; age at onset follows a bimodal distribution with one peak arising in childhood or early adolescence and another in late adolescence or early adulthood [3].

Amongst all psychiatric disorders, OCD is recognized as one of the most serious causes of disability and impaired quality of life in family and social relationships [4–8], and it is associated with considerable economic costs to the individual, healthcare services, and informal caregivers [9]. Serotonergic medications and cognitive behavioural therapy (CBT) with exposure and response prevention techniques (ERP) have been proven to be the first-line treatments [10, 11]. However, as shown by several studies, treatment resistance to first-line treatments is the rule rather than the exception and only about half of patients achieve full remission from OCD symptoms [10, 12–15].

The World Health Organization [16] defined health as a state of complete physical, mental, and social well-being and not merely the absence of disease or infirmity. The Positive Psychology framework [17] developed in the last two decades has highlighted that mental health research also needs to take into account the strengths of the patient and his/her positive outcomes such as satisfaction with life and well-being. This theoretical paradigm shift can allow clinicians and policymakers to make their treatments more effective in the long term to help patients live their lives more meaningfully. Treatment exclusively focusing on OCD symptom reduction may under-recognize the importance of a broader set of quality-of-life and well-being outcomes [17]. Two of these outcomes are sexual functioning and sexual satisfaction [18], which may be associated with improvement in OCD symptoms or recovery or, equally importantly, with a better quality of life despite the presence of OCD symptoms. Greater sexual functioning and sexual satisfaction may be expected to be psychological and relational strengths which can help the person live better despite OCD.

### Sexual dysfunction and sexual satisfaction

Sexuality is one of the facets of quality of life and well-being [19]. A variety of cross-sectional and prospective studies support the view that sexual activity significantly affects overall well-being for both men and women [20–22]. Sexual dysfunctions are characterized by disturbances in sexual desire and in psychophysiological changes associated with the sexual response cycle in men and women [1]. The DSM-5 [1] introduced eight diagnoses: delayed ejaculation, erectile disorder, female orgasmic disorder, female sexual interest-arousal disorder, genito-pelvic pain-penetration disorder, male hypoactive sexual desire disorder, and premature (early) ejaculation, in addition to two non-specific codes (“Other Specified Sexual Dysfunction” and “Unspecified Sexual Dysfunction”). The DSM-5 also specifies substance- or medication-induced disorders, included under Definitions of Sexual Dysfunctions That Can Occur in Men and Women, which identify sexual dysfunctions concerning sexual disorders clearly etiologically related to substance abuse or medication [1].

According to the Consensus Statement of the 4th International Consultation on Sexual Medicine on the epidemiology of sexual dysfunction [23], the most frequent sexual dysfunctions in the general population are desire and arousal dysfunctions for women and premature ejaculation and erectile dysfunction for men. In a nationally representative sample of 1489 UK women [24], 5.8% reported recent sexual dysfunction and 15.5% lifelong sexual dysfunction. Hyposexual desire was the most prevalent recent and lifelong sexual complaint (21.4% and 17.3%, respectively).

Sexual satisfaction is an important ingredient of human sexuality and is considered to be the last stage of the sexual response cycle [25]. There are a number of definitions of sexual satisfaction [26]. It is generally defined as the degree to which an individual is satisfied or happy with the sexual aspect of his or her relationship [27]. One of the most accepted definitions was suggested by Lawrance and Byers [28], who defined it as “an affective response arising from one’s subjective evaluation of the positive and negative dimensions associated with one’s sexual relationship” (p. 268). Sexual satisfaction is associated with better physical and psychological health and well-being [19, 29, 30], with relationship satisfaction, and with better communication with one’s partner [29–31]. Some studies have found a relationship between good sexual functioning and high sexual satisfaction [30].

### Sexual dysfunction and satisfaction in OCD

Sexuality in OCD is an under-recognized topic. One of the first studies conducted in this field [32] showed that about 10% of female patients with OCD reported anorgasmia and 22% had sexual arousal phase problems, whereas 25% of the male patients had lower sexual arousal, 12% premature ejaculation problems and 6% erectile disorder, with 39% of the patients displaying sexual dissatisfaction. More recently, Thakurta and colleagues [33] reported that sexual dysfunction affected 53.33% of a sample of females with OCD. Orgasmic dysfunction was the most frequent dysfunction affecting 20.51% of females, followed by problems of desire at 15.38%. Ghassemzadeh and colleagues [34] found that sexual dysfunction was present in 80.6% of women and 25% of men.

Some evidence from community and clinical research showed that obsessive compulsive symptoms and OCD diagnosis are associated with worse sexual functioning, a higher prevalence of sexual dysfunctions and lower sexual satisfaction. In a national epidemiological study, obsessive compulsive behaviours were one of the strongest predictors of female sexual dysfunction [24]. Kendurkar and Kaur [35] showed that sexual dysfunction was more prevalent among patients with OCD (50%) in comparison with healthy controls (30%).

There are several reasons why patients with OCD may have impaired sexual functioning or satisfaction. A first one may be related to the effects of psychiatric medications [36]. Selective Serotonin Reuptake Inhibitors (SSRIs) may especially delay ejaculation and female orgasm, but also can cause decreased libido and erectile difficulties [37, 38]. Although SSRI-induced sexual side-effects are dose-dependent and generally reversible, high dosages are used in OCD treatment and sexual side-effects can sometimes persist after treatment discontinuation [38]. According to Balon [39], the incidence of SSRI-associated sexual dysfunction is between 30 and 50%. Another clinical aspect which may explain a worse sexual well-being in patients with OCD concerns the content of some symptoms including obsessions related to contamination with diseases, sexual/religious/moral themes, and the possibility of causing harm [40]. Other variables which may contribute to an impaired sexual life may be a poorer physical health [41] or unhealthy behaviours [42].

### Rationale of the present study

Sexual life is a topic under-estimated by clinicians working with OCD. In the international literature, there is no systematic review and meta-analysis about sexual dysfunction and satisfaction in OCD. However, a quantitative summary of the evidence about sexual dysfunction and satisfaction in OCD may have important implications in clinical practice for several reasons. First, it may suggest to practitioners that they investigate sexual life more thoroughly in treatment-seeking patients with OCD with the aim of improving prognosis and treatment response to OCD symptoms, and quality of life. Assessment and treatment of OCD is typically focused on the reduction of clinical symptoms, but little attention is dedicated to those strengths and positive outcomes of the individual that are not directly related to symptoms. Knowledge of sexual life in OCD may also lead to the development of treatment strategies specifically dedicated to sexuality, as no psychotherapeutic protocol is available for OCD associated with sexual dysfunction or low sexual satisfaction. This point appears to be important since poor intimate relations can maintain or be a consequence of symptoms. Preliminary research suggests that a partner’s involvement in therapy (i.e., couple therapy targeting both OCD- and non-OCD-related couple problems/stressors) can enhance symptom reduction and improve couple functioning [43]. Thus, intimate relationships, including a satisfying sexual relationship, could represent a relevant target of OCD treatment. Another important reason to investigate sexual life in OCD is the fact that depression symptoms are very common among patients with OCD [44]. Depression symptoms can be hypothesized to be a moderator of higher sexual dysfunction and lower satisfaction as considerable evidence suggests a significant directional association between depression and sexual dysfunction [45, 46]. Also, some OCD symptoms, such as contamination fears or religious obsessions, and some negative emotions typically associated with OCD, such as disgust and guilt, may have a negative impact on sexuality [47, 48].

Interestingly, some studies showed the presence of sexual dysfunctions independently by SSRIs and/or contamination fear symptoms in female OCD patients [48]. Thus, sexual dysfunction in OCD could represent not just an iatrogenic dimension but a relevant clinical dimension that need a proper assessment and treatment.

### Objectives

The current paper presents a study protocol for a systematic review and meta-analysis aimed to summarize data from primary studies (1) comparing the presence of sexual dysfunction between clinical groups with OCD and non-clinical groups, (2) investigating prevalence of each one of the sexual dysfunctions in patients with OCD, (3) comparing risk for sexual dysfunction in OCD groups with the prevalence in control groups, (4) comparing sexual satisfaction between OCD groups and non-clinical groups, and (5) investigating moderators of sexual dysfunction in OCD groups as compared with control groups. Gender, age, marital status (percentage of married/cohabitant patients with OCD in the study), OCD symptom severity and subtypes, comorbid depressive disorders, comorbid anxiety disorders, concurrent psychiatric medications, comorbid general medical disease, and methodological quality of the studies will be investigated as moderators. It was hypothesized that younger age, male gender, married/cohabitant status, lower OCD severity, lack of depressive disorders, lack of comorbid anxiety disorders, lack of concurrent psychiatric medication, and comorbid medical disease would be associated with a lower risk of sexual dysfunction. A summary of the hypotheses regarding the moderators and the rationale for investigating them is provided in Table 1.

**Table 1:** Summary of moderators

Moderator	Hypothesis	Rationale and evidence for moderators
Age	Younger age is hypothesized to be associated with lower levels of and lower risk for sexual dysfunction, higher sexual satisfaction	Systematic review and community studies showed that younger age is associated with lower prevalence of sexual dysfunction [48–50]
Gender	Male gender is hypothesized to be associated with lower levels of and lower risk for sexual dysfunction, higher levels of sexual satisfaction	Observational studies in clinical samples with OCD showed that females had higher prevalence of sexual dysfunctions than men [34]
Married/Cohabitant status	Married/cohabitant status is hypothesized to be associated with lower levels of and lower risk for sexual dysfunction, higher sexual satisfaction	Married/cohabitant status was found to be associated with lower prevalence of sexual dysfunctions [50]
OCD symptom severity	Higher OCD severity is hypothesized to be associated with lower levels of and lower risk for sexual dysfunction, higher sexual satisfaction	Systematic review showed that higher OCD was associated with worse outcomes in a variety of quality of life domains [5]
Comorbid depressive disorders	Lower percentage of comorbid depressive disorders are hypothesized to be associated with lower levels of and lower risk for sexual dysfunction, higher sexual satisfaction	Observational studies in clinical and non-clinical samples showed that comorbid depressive disorders and/or or symptoms [34, 52]
Comorbid anxiety disorders	Lower percentage of comorbid anxiety disorders are hypothesized to be associated with lower levels of and lower risk for sexual dysfunction, higher sexual satisfaction	Observational studies showed that anxiety disorders are associated with sexual dysfunctions and/or lower sexual satisfaction [53, 54]
Concurrent psychiatric medication	Lack of concurrent psychiatric medication is hypothesized to be associated with lower levels of and lower risk for sexual dysfunction, higher sexual satisfaction	Longitudinal and cross-sectional studies showed that psychiatric medication, specifically antidepressants, are associated with higher levels and prevalence of sexual dysfunctions [36–38]
Comorbid medical disease	Comorbid medical disease is hypothesized to be associated with lower levels of and lower risk for sexual dysfunction, higher sexual satisfaction	Systematic reviews showed that general medical disease is associated with a higher prevalence of sexual dysfunctions [54–56]

## Methods/Design

The systematic review protocol and the procedure used for meta-analysis have been presented according to the PRISMA-Protocol (PRISMA-P) [58] and were registered in PROSPERO on CRD42019132264. Any amendments will be updated on PROSPERO and documented accordingly.

### Eligibility criteria

Studies will be included if (a) they are conducted on adult clinical groups aged between 18 and 65 years old with a current primary diagnosis of OCD; (b) OCD has been diagnosed through a clinical diagnosis or using a standardized diagnostic clinician-administered interview, e.g., the *Structured Clinical Interview for Axis I Disorders* (SCID-I) [59] based upon a standardized diagnostic international classification system (e.g., any version of the DSM or the ICD) or a validated self-report measure of OCD symptoms (i.e., the reliability values are well-established) such as the Yale-Brown Obsessive Compulsive Scale (Y-BOCS) [60]; (c) they define sexual dysfunction according to a standardized diagnostic classification system (e.g., any version of the DSM or the ICD) and investigate sexual dysfunction according to the criteria provided by those systems, or investigate sexual satisfaction; (d) they used a clinician-administered interview or a self-report questionnaire with known psychometric properties (i.e., reliability values) to assess sexual dysfunction or sexual satisfaction (overviews of the eligible measures of sexual dysfunction or satisfaction are presented in Tables 2 and 3); (e) they used any type of research design; (f) they measured the presence of any type of sexual dysfunction or focused on a specific type of sexual dysfunction; and (g) they report the necessary data on effect sizes (please, see the “Data pooling and meta-analysis” section further on for effect size calculation) or the authors are willing to provide the necessary data when contacted if such data are missing in the study paper.

**Table 2:** Sexual dysfunction measures which will be included in the systematic review

Eligible measures to assess sexual dysfunction	Coding of broad categories of sexual dysfunctions (DSM-5) (an asterisk indicates that the measure contains a score on the specific dysfunction)								Coding of gender-based categories (an asterisk indicates that the measure contains a dysfunction score specific to gender)		
	General sexual dysfunction	Delayed ejaculation	Premature ejaculation	Erectile disorder	Male hypoactive sexual desire disorder	Female orgasm disorder	Female sexual interest-arousal disorder	Genito-pelvic pain-penetration disorder	Male dysfunction	Female dysfunction	Both sexes
Arizona Sexual Experience Scale (ASEX [61];)		*	*	*	*	*	*				*
Changes in Sexual Functioning Questionnaire (CSFQ [62];)	*	*	*	*	*	*	*				*
Derogatis interview for sexual functioning (DISF/DISF-SR [63];)	*	*	*	*	*	*	*				*
Derogatis Sexual Functioning Inventory [64]	*	*	*	*	*	*	*				*
Female Sexual Function Index (FSFI [65];)	*					*	*	*		*	
International Index Erectile Function [66]	*	*	*	*	*				*		
Israeli Sexual Behavior Inventory [67]	*		*	*	*	*	*	*			*
Massachusetts General Hospital Sexual Functioning Questionnaire (MGH [68];)	*										*
Sexual Activity Questionnaire (SAQ [69];)						*				*	
Sexual Arousability Inventory (SAI [70];)							*			*	
Sexual Interaction Inventory [71]	*										*
Sexual Interaction System Scale (SISS [72];)	*										*

**Table 3:** Sexual satisfaction measures which will be included in the systematic review

Eligible measures to assess sexual satisfaction	
Arizona Sexual Experience Scale (ASEX [61];)	
Golombok Rust Inventory of Sexual Satisfaction (GRISS [73];)	
Female Sexual Function Index	
Hudson Index of Sexual Satisfaction [74]	
Interpersonal Exchange Model of Sexual Satisfaction (IEMSS [75];)	
International Index Erectile Function [66]	
Pinney Sexual Satisfaction Inventory [76]	
Sexual Activity Questionnaire (SAQ [69];)	
Sexual Interaction Inventory [71]	
Sexual Satisfaction Scale for Women (SSS-W [77];)	
Whitley Sexual Satisfaction Inventory [78]	

Studies where patients had concurrent psychosis, bipolar disorders, neurological disorders, or mental retardation will be excluded at full text review. This methodological choice aims to allow us to analyze sexual dysfunction/satisfaction in patients with OCD by excluding the biasing effects of such major psychiatric/neurological disorders which can significantly impact sexual functioning.

No language restriction will be applied. Studies will be included whether they used inpatients or outpatients. The presence of concurrent treatment, either pharmacological or psychotherapeutic, will not be an exclusion criterion. If some of the patients are on concurrent medication, the percentage of those on medication will have to have been reported and the type of medication specified. No restriction on publication dates will be applied. Studies using patients with a lifetime diagnosis of OCD or with subclinical or remitted OCD symptoms will not be excluded. Studies where patients had concurrent psychosis, bipolar disorders, neurological disorders, or mental retardation will not be excluded.

### Information sources and search procedure

The search will be conducted during the second week of December 2019. Studies will be identified by conducting a systematic search of electronic databases using Medical Subject Headings (MeSH terms) and keywords related to “Obsessive Compulsive Disorder” which will be combined through the Boolean operator “AND” with MeSH terms and keywords related to “Sexual Dysfunction” or “Sexual Satisfaction”. MeSH terms were created by using the PubMed MeSH on Demand Tool which allowed us to identify relevant MeSH terms. The search procedure will be conducted using the databases Scopus, PubMed, EMBASE, PsycINFO, CINAHL, and the Cochrane Library. An overview of the electronic search strategy is provided in Table 4. The search based on electronic databases will be piloted by one of the reviewers (AP) through a preliminary search to ensure all relevant keywords have been captured.

**Table 4:** Electronic search procedure

Electronic databases	Search terms (MeSH and keywords)
	MeSH: “Obsessive Compulsive Disorder” Keywords: Obsessive Compulsive Disorder Neurosis Obsessions Compulsions
Scopus PubMed EMBASE PsycINFO CINAHL Cochrane Library	MeSH: “Sexual Dysfunction” Keywords: Frigidity Hypoactive Sexual Desire Disorder Orgasmic Disorder Psychosexual Disorders Psychosexual Dysfunctions Sexual Arousal Disorder Sexual Aversion Disorder Sexual Functioning Sexual Disorders
	MeSH: “Sexual Satisfaction” Keywords: Sexual Satisfaction Sexual behaviour Sexual gratification Orgasm Sexual pleasure

Note: *MeSH* Medical Subject Heading

A search will be conducted also on the following major clinical trials registries: ISRCTN Registry, EU Clinical Trials Register (EU-CTR), ClinicalTrials.gov, Clinical Research Information Service (CRiS), Australian New Zealand Clinical Trials Registry (ANZCTR), and Chinese Clinical Trial Registry (ChiCTR).

In addition, to identify any further published or unpublished studies, all the authors of the studies included will be contacted. Reference sections of included studies will be checked. Conference proceedings will be hand-searched from inception for abstracts, papers, or posters presented at the following international scientific societies relevant to research on OCD: *American Psychiatry Association*, *American Psychological Association*, *Anxiety and Depression Association of America*, *Association for Behavioral and Cognitive Therapies*, *European Association of Behavioural and Cognitive Therapies*, *European Association of Psychiatry*, *European Association of Psychology*, *British Psychological Society*, *Royal College of Psychiatrists*, *British Association of Behavioural and Cognitive Psychotherapy*, *International Academy of Sex Research*, *International Obsessive Compulsive Disorder Foundation*, *International Society for Sexual Medicine*, *European Society for Sexual Medicine*, *Sexual Medicine Society of North America*, *International College of Obsessive Compulsive Spectrum Disorders*. This search will be carried out independently by the two reviewers by accessing the websites of these scientific societies. For any conference abstracts/papers/posters judged as potentially eligible, the corresponding author will be contacted to request further information to judge whether or not the work can be included and/or further data should be requested to perform the meta-analysis. Eligible theses and doctoral dissertations will be searched and identified by the two independent reviewers who will run the same queries using the same keywords on the Open Access Theses and Dissertations website.

The review process including study selection, data extraction, and risk of bias assessment will be performed through the software Covidence.

### Selection of studies

Studies will be assessed and screened by two independent reviewers (AP, DD) in three stages using inclusion/exclusion criteria. During the first stage, studies will be assessed independently by the reviewers with regards to inclusion criteria after reading the title. Then, the reviewers will meet to compare their selections. During the abstract selection stage, the two reviewers will independently assess each of the retained studies by reading the abstract and again they will meet to compare their selections. During both these stages (exclusion by title and by abstract), only studies on which both reviewers are in complete agreement on exclusion will be excluded. On the contrary, studies will be retained if there is disagreement between the reviewers on inclusion or exclusion. During the final stage, studies will be assessed independently by the two reviewers by assessing the full text of the paper. Potential discrepancies on inclusion or exclusion at this stage and their reasons will be discussed and resolved in a meeting with two other independent reviewers (UA, DP) to obtain an agreed-upon number of included studies. Between-reviewer agreement on inclusion will be calculated by the Kappa index [79]. After this meeting, the decision regarding the inclusion of the study will be made by another independent reviewer (DM) in a final meeting with the other reviewers.

### Data extraction

All information will be extracted from each of the included studies by two independent reviewers (AP, DD) and inserted into an Excel worksheet after an initial pilot using three included studies. Table 5 provides information on what will be extracted and coded from the primary studies. A third independent reviewer (DP) not involved in the extraction process will check the correctness of the data inserted in the worksheet. After data insertion is completed, potential discrepancies in the data extracted by the two reviewers will be discussed at a meeting between the reviewers who conducted the data extraction and the third independent reviewer.

**Table 5:** Information extracted from the primary studies and coding procedure

Information extracted	Coding	Moderator
Title of the paper	Full title of the paper	No
First author name	First author’s last name	No
Publication date	Publication date of the paper	No
Language of the paper	Language in which the paper is written	No
Publication on a peer-review journal	“Yes”, “No”	No
Publication type	“Published on a journal”, “Conference paper”, “Thesis/doctoral dissertation”	No
Country where the study was conducted	Name of the country	No
Participants’ inclusion criteria	Quote the inclusion criteria reported in the study paper	No
Participants’ exclusion criteria	Quote the exclusion criteria reported in the study paper	No
Total sample size in the study		No
Participants with OCD	Number of clinical participants with OCD	No
Control participants	Number of control participants	No
Type of control participants	“Undergraduates”, “Community individuals”	No
Matched controls	“Yes”, “No”. If Yes, specify if match was made on age or gender or both	No
Age	Total study mean age and standard deviation	Yes
Females	Total percentage of females	Yes
Married/cohabitant patients	Total percentage of married/cohabitant patients	Yes
Employed patients	Percentage of employed patients	No
OCD symptom severity	Mean Y-BOCS scores	Yes
Research design	“Cross-sectional case-control”, “Longitudinal”	No
OCD diagnosis	Diagnostic criteria used to establish OCD diagnosis	No
Instrument(s) used to establish OCD diagnosis	Acronym of the instrument(s)	No
Instrument(s) used to assess sexual dysfunction	Acronym of the instrument(s)	No
Type of instrument(s) used to assess sexual dysfunction	“Clinician-administered interview”, “Self-report questionnaire”	No
Sexual dysfunction diagnosis	Diagnostic criteria used to define sexual dysfunction	No
Sexual dysfunction type	Type of sexual dysfunction according to the classification system used in the study	No
Sexual satisfaction	Criteria used to define sexual satisfaction	No
Instrument(s) used to assess sexual satisfaction	Acronym of the instrument(s)	No
Age at OCD onset	Mean age at OCD onset in the study	No
Duration of OCD	Study mean duration of OCD symptoms in months	No
Patients on concurrent psychiatric medication	Percentage of patients on concurrent psychiatric medication	Yes
Types of prescribed psychiatric medication	“Atypical antipsychotics”, “Antidepressants”, “Anxiolytics”	No
Types of antidepressants	Generic name of the antidepressant type	No
Types of antipsychotics	Generic name of the antipsychotic type	
Presence of concurrent psychotherapeutic treatment	“Yes”, “No”	No
Clinical population	“Outpatient”, “Inpatient”	No
Patients with sexual obsessions/compulsions	Percentage of patients with sexual obsessions/compulsions	No
Patients with contamination obsessions and/or washing compulsions	Percentage of patients with contamination obsessions and/or washing compulsions	No
Strategies used to recruit clinical participants	Quote the strategies reported in the study paper	No
Strategies used to recruit controls	Quote the strategies reported in the study paper	No
Setting where clinical participants were recruited	Quote the setting where patients were recruited	No
Measure(s) of depressive symptoms	Acronym of the measure(s)	No
Measure(s) of anxiety symptoms	Acronym of the measure(s)	No
Comorbid depressive disorders	Percentage of patients with comorbid depressive disorders	Yes
Comorbid anxiety disorders	Percentage of patients with any comorbid anxiety disorders (if different anxiety disorders are reported a mean percentage of any anxiety disorders in the study is calculated)	Yes
Comorbid eating disorders	Percentage of patients with comorbid eating disorders	No
Comorbid personality disorders	Percentage of patients with comorbid personality disorders	No
Comorbid alcohol/substance abuse/dependence	Percentage of patients with comorbid alcohol/substance abuse/dependence	No
Comorbid general medical disease	Percentage of patients with comorbid general medical disease	Yes
Study methodological quality	Score on the Newcastle-Ottawa Scale	Yes

### Evaluation of study quality

The quality of each study will be independently evaluated by two reviewers (AP, DD) using the Newcastle-Ottawa Quality Assessment Scale [80]. The final decision regarding the quality score to assign to each study will be made by a third independent reviewer (DM) during a meeting with the two quality assessors. This tool assigns a maximum score of nine: four points regarding inclusion criteria for cases and controls (definition of cases, selection of cases, definition of controls, selection of controls), two points regarding the comparability criteria of cases and controls according to study design and statistical analysis (comparability in terms of age and in terms of gender), and three points for exposure verification criteria of cases and controls (exposure verification, same method of verification, no response point). Studies scoring nine are classified as high quality, those scoring seven or eight as medium quality, and those scoring less than seven as low quality. Disagreement in score attribution between the two authors will be settled by discussion.

### Data pooling and meta-analysis

#### Summary measures

If there is insufficient data for a specific outcome, only a systematic review will be performed. Data will be pooled from studies assessing the mean differences between OCD groups and control groups regarding the levels of sexual dysfunction, sexual satisfaction, and risk of sexual dysfunction. If there is sufficient data, a random-effect meta-analysis will be conducted using the software *Comprehensive Meta-Analysis, CMA version 2.00* [81]. For all the analyses, the *p* value will be set at 0.05. Random-effect models assume that included studies are drawn from populations of studies that systematically differ from each other [81]. According to these models, effect sizes extracted from included studies differ not only because of random error within studies (as in fixed-effect models), but also because of true variation in effect sizes from one study to another. Summary measures will consist of effect-size indexes related to (1) levels of sexual dysfunction, (2) levels of sexual satisfaction, and (3) risk of sexual dysfunction in OCD groups as compared to control groups. Effect-size indexes related to levels of sexual dysfunction and levels of sexual satisfaction in OCD as compared to controls will be calculated using the following formula proposed by Cohen [82]: *d* = (*M*_CASE_ − *M*_CONTROL_)/*SD*_COMBINED_, where *M*_CASE_ and *M*_CONTROL_ represent the means of the OCD and control groups, respectively, and *SD*_COMBINED_ is the combined standard deviation. If a study does not include a control group, an estimate of the general population mean for a particular sexual dysfunction will be computed, based on current prevalence studies or normative mean (and standard deviation), and it will be compared with the observed data on the OCD group extracted from the study to generate a standardized mean difference.

Effect-size indexes related to risk of sexual dysfunction in OCD as compared to controls will be calculated by calculating the effect-size index based on the following formula: Relative Risk Ratio = (*A*/*n*_1_)/(*C*/*n*_2_), where *A*/*n*_1_ represents the probability of sexual dysfunction in OCD groups and *C*/*n*_2_ the probability of sexual dysfunction in control groups. For each study, a mean effect-size index will be calculated by pooling the effect-size indexes related to all sexual dysfunctions assessed by the study. In addition, for each study, an effect-size index will also be calculated for every single sexual dysfunction. A value lower than 1 suggests that the incidence of sexual dysfunction is lower in OCD groups than in control groups, while a value higher than 1 indicates that it is higher in the OCD than in the control groups.

The score of each index will be weighted using the following correction formula: *W*_zr_ = 1/*SE*^2^, where *SE*^2^_zr_ is the standard error of the effect-size index calculated for each study. Using Cohen’s model, effect-size indexes greater than or equal to 0.80 are considered high, indexes in the range of 0.80–0.50 moderate, and indexes less than or equal to 0.20 low. Hedges’ correction for small sample bias will be applied [83].

Effect sizes for each sexual dysfunction will be coded by two independent reviewers who will code the measures according to the system presented in Table 2, and a third independent reviewer will have the final word if discrepancy emerges.

#### Expert consultation

To assess whether the evidence from the meta-analysis on sexual dysfunction/satisfaction are clinically meaningful, a Delphi method will be followed by presenting the evidence found through meta-analysis to a panel of five experts. They will be selected according to the following criteria: (a) being associate professor or higher in the field related to this review topic (i.e., clinical psychology, sexology, psychiatry), (b) editorial board membership for a journal related to OCD and/or sexology, (c) having published at least 15 papers on peer-review journals about OCD or sexology. Experts will be contacted and invited to participate by email by one of the reviewers (DD) who will act as moderator of the panel. The first consultation will be dedicated to presenting to each expert the issue addressed by the review and the meta-analysis evidence. In a second consultation, each expert will be asked separately the following two questions: (1) “Do these findings fit with your clinical experience?” (2) “What is their strengths for clinical practice with OCD patients?” After this session, the statement of each expert will be recorded verbatim by the moderator and sent to the other experts who will be given time to complete or refine their statements. A last session will be dedicated to a final comparison of the experts’ input and the meta-analysis evidence by the moderator and another reviewer (AP).

#### Publication bias

To assess the likelihood that effect sizes have been subject to publication bias, two procedures will be used: Duval and Tweedie’s trim and fill procedure and a visual inspection of the funnel plot [81]. A funnel plot is a scatter plot in which the effect sizes computed from the included studies are plotted on the horizontal axis against an indicator of study precision, the Standardized Error, on the vertical axis [81]. In the absence of bias, the graph resembles a symmetrical inverted funnel because the effect sizes derived from smaller studies scatter more widely at the bottom of the graph, with the spread narrowing as precision increases among larger studies. If there is publication bias because smaller studies reporting no significant effect sizes remain unpublished, then the funnel plot appears asymmetrical [81].

#### Inconsistency analysis

To verify heterogeneity in effect sizes, the prediction intervals and the *Q* index will be calculated [84, 85]. The *Q* index is calculated by summing the squared deviation of each study’s effect estimate from the overall effect estimate, while weighting the contribution of each study by its inverse variance [83]. In the hypothesis of homogeneity among effect sizes, the *Q* statistic follows a chi-square distribution with *k* − 1 degrees of freedom, *k* being the number of studies.

#### Moderator analysis

If inconsistency between effect sizes is found, simple regression analyses by weighted least squares will be performed to investigate whether any of the following variables can moderate the effect sizes: (a) age, (b) gender, (c) marital status (coded as the total percentage of married/cohabitant patients), (d) OCD symptom severity (measured by the Y-BOCS scores), (e) concurrent psychiatric medication, (f) comorbid depressive disorders, (g) comorbid anxiety disorders, (h) comorbid general medical disease, (i) study methodological quality coded as NOS scores.

#### Sensitivity analysis

To further account for inconsistency, sensitivity analyses will be performed by calculating the effect sizes only on studies using patients without concurrent psychiatric medication and on those excluding patients with comorbid general medical disease.

## Discussion

Sexuality is an important aspect of relational life and has a key role in the quality of life and well-being [19, 21]. However, sexual functioning and sexual satisfaction are rarely considered aspects of the life of patients with OCD in clinical practice because OCD assessment and treatment typically focus on the reduction of symptoms; little attention is dedicated to strengths and positive outcomes of individuals with this condition. Studies on the general population showed that obsessive compulsive tendencies are associated with sexual dysfunction or lower sexual satisfaction [24]; higher prevalence of sexual dysfunctions and lower sexual satisfaction were found in patients with OCD as compared with community or healthy controls [35].

There is a need for a summary of the evidence about the presence of sexual dysfunction and the degree of sexual satisfaction in patients with OCD. In the literature, there is no systematic review addressing these two points. Several reasons may be considered to explain why patients with OCD may be expected to have impaired sexual functioning or lower satisfaction. A first point is related to the presence of concurrent psychiatric medications [36] since SSRIs may delay ejaculation and female orgasm and can also cause decreased libido and erectile difficulties [37, 38].

Focusing assessment also on sexuality in OCD may improve prognosis, treatment response to OCD symptoms, and quality of life. Increased knowledge of sexuality in OCD may suggest the introduction of treatment strategies dedicated to sexuality. Recent research has shown that a partner’s involvement in therapy (i.e., couple therapy targeting both OCD and non-OCD related couple problems and stressors) can enhance symptom reduction and improve couple functioning [43]. Another important reason to investigate sexual life in OCD is the fact that depressive symptoms are very common among patients with OCD [86].

This systematic review also has the strengths of independent study selection and data extraction, a search strategy based on the identification of published and unpublished studies and data including theses and doctoral dissertations, and, lastly, an evaluation of the study’s methodological quality. The sensitivity analysis of the effect sizes which exclude studies with patients on psychiatric medications or with general medical disease will allow us to more clearly investigate the relationship between OCD and sexuality by excluding factors which could potentially confuse the association between OCD and sexual dysfunction/satisfaction.

Potential limitations will regard a small number of studies in the literature and the heterogeneity of the studies in terms of the instruments used to assess sexual dysfunction and sexual satisfaction and of the definitions used to conceptualize sexual dysfunction. In addition, the cross-sectional design of the studies to be included will not allow us to draw causal conclusions about the relation between OCD and sexual dysfunction or impaired sexual satisfaction. Another limitation of our protocol is the lack of a focus on sexual preferences and sexual orientation (i.e., heterosexual, homosexual, bisexual orientation). This represents an interesting point which needs for a broader investigation in future reviews.

Future works including randomized controlled designs should focus on sexual functioning/satisfaction in patients with OCD after and/or during the administration of medications. It may be interesting to explore which types of psychiatric medications are associated with a negative/positive sexual functioning to orient clinical practice. It should also be investigated which patients’ characteristics may be associated with the effects of medications (e.g., comorbidities, symptom subtypes, demographic factors).

The investigation of the evidence regarding sexual functioning/satisfaction in patients with OCD may advance research on quality of life in this clinical population. Further work should investigate whether standard treatments for OCD, i.e., CBT, produce an improvement in sexual well-being and whether such an improvement correlates with an increase in the general level of quality of life. It may be hypothesized that some recently developed treatment protocols for OCD may produce positive effects on sexual life such as mindfulness-based interventions, since they are aimed to improve awareness of body and internal/external experiences [87]. In addition, future research should be dedicated to the integration of strategies of sexual therapy in the standard treatment of patients with OCD.

Another gap to be addressed in future research may be the investigation of the clinical variables which might explain why an impaired sexual functioning can be observed in patients with OCD. This gap might be investigated through the comparison of sexual outcomes between patients with OCD and patients with other obsessive compulsive spectrum disorders involving a difficult relation with body, such as body dysmorphic disorders or skin picking disorders [88].

In conclusion, this is a protocol of the first systematic review of sexual dysfunction and sexual satisfaction in patients with OCD. A clear summary of the evidence on these topics may support clinical practice highlighting the importance of the assessment of sexuality in OCD and suggesting the use of therapeutic strategies dedicated to sexuality in OCD with the aim of improving patients’ quality of life.

## Data Availability

Not applicable

## Abbreviations

ASEX: Arizona Sexual Experience Scale
CBT: Cognitive behavioural therapy
CSFQ: Changes in Sexual Functioning Questionnaire
DISF/DIS-SR: Derogatis interview for sexual functioning
FSFI: Female Sexual Function Index
GRISS: Golombok Rust Inventory of Sexual Satisfaction
IEMSS: Interpersonal Exchange Model of Sexual Satisfaction
MGH: Massachusetts General Hospital Sexual Functioning Questionnaire
OCD: Obsessive Compulsive Disorder
PRISMA-P: PRISMA-Protocol
SAI: Sexual Arousability Inventory
SAQ: Sexual Activity Questionnaire
SCID-I: Structured Clinical Interview for Axis I Disorders
SISS: Sexual Interaction System Scale
SSRIs: Selective Serotonin Reuptake Inhibitors
SSS-W: Sexual Satisfaction Scale for Women
Y-BOCS: Yale-Brown Obsessive Compulsive Scale
